# Early assessment with ^18^F-2-fluoro-2-deoxyglucose positron emission tomography/computed tomography to predict short-term outcome in clear cell renal carcinoma treated with nivolumab

**DOI:** 10.1186/s12885-019-5510-y

**Published:** 2019-04-02

**Authors:** Tadashi Tabei, Noboru Nakaigawa, Tomohiro Kaneta, Ichiro Ikeda, Keiichi Kondo, Kazuhide Makiyama, Hisashi Hasumi, Narihiko Hayashi, Takashi Kawahara, Koji Izumi, Kimito Osaka, Kentaro Muraoka, Jun-ichi Teranishi, Yasuhide Miyoshi, Yasushi Yumura, Hiroji Uemura, Kazuki Kobayashi, Tomio Inoue, Masahiro Yao

**Affiliations:** 1 Department of Urology, Yokohama City University Graduate School of Medicine, 3-9 Fukuura, Kanazawa, Yokohama, Kanagawa Japan; 2 Department of Urology, Yokosuka Kyosai Hospital, 1-16 Yonegahama-Dori, Yokosuka, Kanagawa Japan; 3 Department of Radiology, Yokohama City University Graduate School of Medicine, 3-9 Fukuura, Kanazawa, Yokohama, Kanagawa Japan; 4 Department of Urology, Yokohama Minami Kyosai Hospital, 1-21-1 Mutsuura Higashi, Kanazawa, Yokohama, Kanagawa Japan

**Keywords:** Carcinoma, Renal cell, Positron-emission tomography, Computed tomography, Nivolumab, Antineoplastic agents

## Abstract

**Background:**

We reported previously the usefulness of 18F-2-fluoro-2-deoxyglucose positron emission tomography/computed tomography (FDG-PET/CT) to predict prognosis of renal cell carcinoma (RCC) treated with molecular targeted agents. Herein we describe a preliminary research of nine patients who underwent FDG-PET/CT before and after initiation of nivolumab.

**Methods:**

Patients with metastatic RCC who were treated by nivolumab from October 2016 to March 2017 were enrolled in this study. All patients underwent FDG-PET/CT at baseline and 1 month as a first response assessment, and contrast-enhanced or non-contrast-enhanced CT scan at 4 month as a second response assessment. Logistic regression analysis was performed to assess the association of potential predictors, including age, gender, baseline diameter, baseline maximum standardized uptake value (SUVmax), lung or not lung metastasis, elevation of SUVmax at 1st assessment, and decrease in diameter at 1st assessment with the response at 2nd assessment (decrease in the diameter ≥ 30% or not).

**Results:**

There were 9 patients and 30 lesions. Mean days of first assessment with FDG-PET/CT and second assessment by CT scan from initiation of treatment were 32.3 ± 6.4, 115.5 ± 14.9, respectively. Lesions whose diameter decreased ≥30% at second assessment were defined as responding, and lesions whose diameter did not decrease ≥30% were defined as non-responding. There were 18 responding lesions, and 12 non-responding lesions. We compared change in diameter and SUVmax at first assessment with FDG-PET/CT, respectively. All lesions with decreased diameter and elevated SUVmax at first assessment with FDG-PET/CT showed responding at second assessment by CT scan, while most lesions with increased diameter and declined SUVmax at first assessment showed non-responding at second assessment. The multivariate logistic regression analyses revealed that only the elevation of SUVmax at 1 month was an independent predictor (*P* = 0.025, OR: 13.087, 95%CI: 1.373–124.716).

**Conclusion:**

Our findings suggest that the early assessment using FDG-PET/CT can be effective to predict the response of RCC to nivolumab. However, larger prospective studies are needed to confirm these preliminary results.

**Trial registration:**

Registered in University Hospital Medical Information Network in JAPAN [UMIN0000008141], registration date: 11 Jun 2012.

## Background

Approximately 150,000 patients around the world are assumed to die of kidney cancer each year [1]. There were 34,700 kidney-cancer-related deaths in the European Union in 2012 [2]. Renal cell carcinoma (RCC) accounts for 2–3% of all cancer cases [3]. About 30% of patients have metastatic lesions at the time of diagnosis, and an additional 20–40% of patients develop metastases despite curative treatment such as radical nephrectomy [4, 5].

Tyrosine kinase inhibitors (TKIs) or mammalian target of rapamycin (mTOR) inhibitors were two major innovative drugs in the history of RCC treatment [6–8]. In most malignant neoplasms, changes in tumor burden are regarded as important surrogate markers for survival. However, there are cases in which growth of RCC ceases, as if the cancer entered a period of dormancy after initiating those drugs. It is challenging to decide whether current therapy should be continued for such patients. Unfortunately, we do not have any practical markers that can reflect the biological activity of RCC.

Our group previously reported the usefulness of ^18^F-2-fluoro-2-deoxyglucose positron emission tomography/computed tomography (FDG-PET/CT) in predicting prognosis in patients on systemic therapy with TKI [9, 10] or mTOR inhibition [11]. These reports demonstrated decreasing maximum standardized uptake value (SUVmax) after treatment with those drugs was predictive of a favorable prognosis. Some studies from other institutions have reported the relationship between SUVmax and prognosis as well [12–14]. Therefore it is reasonable to think that decreasing SUVmax indicates a decrease in a cancer’s biological activity.

Immune checkpoint inhibitors are novel anti-tumor agents, including an anti-cytotoxic T-lymphocyte-associated protein 4 (anti-CTLA4) monoclonal antibody, anti-programmed death 1 (anti-PD1) monoclonal antibody, and anti-programmed death ligand 1 (anti-PDL1) monoclonal antibody. Today these agents are available for various malignancies including malignant melanoma, non-small cell lung cancer (NSCLC), urothelial carcinoma, and head and neck cancer [15–19]. In 2015, a phase 3 randomized study (CheckMate025) demonstrated the superior effectiveness of nivolumab, an immunoglobulin (Ig)-G4 subclass programmed-death-1 inhibiting antibody, compared to everolimus, for patients with advanced clear cell RCC who had received previous antiangiogenic treatment [20]. Following this study, nivolumab was recommended as 2nd line therapy for metastatic RCC in European Association of Urology [21] and the National Comprehensive Cancer Network [22] guidelines. Immune checkpoint inhibitors were thought to improve the capability of cytotoxic T-lymphocytes under the immunosuppressive conditions induced by malignancies to mount an effective response. The novel antitumor mechanism of immune checkpoint inhibitors showed us a new and characteristic response called “pseudoprogression.” The mechanism of “pseudoprogression” was speculated to be the result of infiltration of activated lymphocytes or other inflammatory cells that induced immune modulators that sometimes make tumors temporarily larger. These phenomena led us to assume that the FDG accumulation during treatment by nivolumab might be different from that seen with treatment by molecular targeted drugs. Therefore, it might not be valid to interpret findings in FDG-PET/CT when evaluating the response to nivolumab in the same way as when evaluating the response to other modes of therapy. However, any studies investigating that subject in RCC are not known so far. Herein we describe preliminary reports of nine patients who underwent FDG-PET/CT before and after initiation of nivolumab.

## Methods

This study was a phase II pilot study to investigate the association between the FDG accumulation change and the response of RCC to nivolumab, which had not been investigated previously. The patients with RCC who were planned to be treated by nivolumab in Yokohama City University from October 2016 to March 2017 were enrolled in this study with the written consent. The 30 RCC lesions were planned to be investigated and the enrollment was closed when the targeted lesions become more or 30, in order to minimize the number of research subjects.

All patients underwent FDG-PET/CT at baseline and 1 month as a first response assessment, and contrast-enhanced or non-contrast-enhanced CT scan at 4 month as a second response assessment. All patients received 3 mg/kg of nivolumab intravenously every 2 weeks. Patients with uncontrolled diabetes mellitus (FBS > 150mg/dl) or other known malignancy were excluded from this study. Nivolumab was continued unless disease progression or intolerable adverse events occurred. We measured diameters and SUV max of each measurable lesion, defined as > 10 mm in longest diameter or a lymph node > 15 mm in shortest diameter according to RECIST ver1.1 [23].

### Statistical analysis

Logistic regression analysis was performed to assess the association of potential predictors, including age, gender, baseline diameter, baseline SUVmax, lung or not lung metastasis, elevation of SUVmax at 1st assessment (Yes or No), and decrease in diameter at 1st assessment (Yes or No) with the response at 2nd assessment (responding or not responding). All statistical analyses were carried out with commercial software (SPSS® version19, SPSS Inc., Chicago, IL, USA) with advice by the statistician who had enough experience.

### Imaging with FDG-PET/CT

FDG-PET/CT imaging was performed as described in our previous study [9].

Patients fasted for at least 6 h prior to intravenous injection of ^18^F FDG. PET/CT images were obtained using a PET/CT system (Aquiduo 16; Toshiba Medical Systems, Tokyo, Japan). A low-dose non-contrast CT scan was acquired first and used for attenuation correction. Images were acquired from the top of the head to the mid-thigh in 3-dimensional mode for 2 min per bed position 60 min after intravenous injection of 2.5 MBq/kg of ^18^F FDG. After PET acquisition, CT scan was performed with a 2-mm slice thickness, 120 kV, 400 mA, 0.5 s/tube rotation, from the top of the head to the mid-thigh, with breath holding. Images were reconstructed by attenuation-weighted ordered-subset expectation maximization (OSEM) (four iterations, 14 subsets, 128 × 128 matrix, with 5-mm Gaussian smoothing). The standardized uptake value (SUV) was calculated either pixel-wise or over a region of interest (ROI) for each image of a dynamic series at time points t as the ratio of tissue radioactivity concentration (MBq/kg) at time t, c(t), and injected dose (MBq) at the time of injection (*t* = 0) divided by body weight (kg). SUV = c(t) / [injected dose (t_0_) / body weight].

## Results

There were nine patients and 30 lesions. The patients’ characteristics are shown in Table 1. All patients were diagnosed as clear cell RCC by prior nephrectomy or biopsy; metastatic or recurrent lesions were confirmed by computed tomography with or without contrast. Three patients had had metastatic lesions at diagnosis while six had developed recurrent disease after treatment with curative intent. There were seven men and two women. Their mean age was 68.8 ± 3.9 years old. The most frequent sites of metastatic lesions were lung (37%) and lymph nodes (17%) (Table 2). The mean days of first assessment with PET/CT or second assessment by CT scan from initiation were 32.3 ± 6.4, 115.5 ± 14.9, respectively. Changes in tumor diameter and SUVmax in each lesion are shown in Table 2. There were five patients in partial remission (PR), three with stable disease (SD) and one with progressive disease (PD) according to RECIST ver1.1.

**Table 1:** Patients’ characteristics

Pt	Age	Histological Type	T stage	Tumor Grade	KPS	Prior Therapy						
						1st	2nd	3rd	4th	5th	6th	7th
1	60s	clear	3	G2	90	SU	EVR	AXT	PAZ	TEM		
2	70s	clear	1a	G1	100	INF	SFN	EVR	AXT			
3	60s	clear	2a	G1	100	SU	AXT	EVR	PAZ	SFN		
4	60s	clear	4	G1	100	SU	AXT	PAZ				
5	60s	clear	1b	G2	100	INF	SFN	EVR	AXT	SU	TEM	PAZ
6	60s	clear	3a	G2	100	INF	SFN					
7	60s	clear	1b	G1	90	SFN	EVR	AXT				
8	60s	clear	3a	G2	100	SFN	AXT	EVR	SU			
9	70s	clear	1b	G3	90	SFN	EVR					

*KPS* Karnofsky performance status, *SU* Sunitinib, *SFN* Sorafenib, *EVR* Everolimus, *AXT* Axitinib, *PAZ* Pazopanib, *TEM* Temsirolimus, *INF* Interferon-alpha

**Table 2:** Diameter and maximum standardized uptake value (SUVmax) of each lesion at each timepoint in treatment with nivolumab

Patient	Site of Lesion	Baseline		1st assessment			2nd assessment	
		Diameter (mm)	SUVmax	Diameter (mm)	SUVmax	Response	Diameter (mm)	Response
1	kidney (primary)	63	7.5	65	3.7	SD	62	SD
	LN	60	7.6	61	6.3		60	
	lung	10	1.5	10	1.6		7	
2	bone	49	5.4	50	5.0	SD	54	PD^a^
	lung	27	3.5	29	3.3		33	
	lung	12	2.8	14	2.2		14	
3	lung	39	8.8	28	8.5	SD	18	PR
	subcutaneous	13	1.9	10	1.0		4	
4	bone	65	5.9	57	4.7	SD	60	SD
	adrenal	45	3.7	45	3.6		60	
	bone	41	3.4	38	3.3		35	
5	subcutaneous	161	5.1	151	6.7	SD	90	PR
	adrenal	38	5.1	39	6.3		21	
	liver	33	4.5	38	5.4		23	
	LN	15	3.4	12	3.4		0	
	lung	12	5	12	5.4		12	
	muscle	45	4.2	45	5.6		31	
	bone	15	2.8	14	3.5		0	
	subcutaneous	15	2.1	14	3.7		0	
6	lung	18	4.1	19	2.0	SD	8	PR
	lung	23	3.5	20	6.3		11	
	adrenal	28	2.5	28	5.1		21	
7	LN	15	3.3	15	2.9	SD	6	PR
	lung	36	4.8	36	4.2		17	
8	lung	22	5.1	17	6.1	SD	10	SD
	lung	17	2.2	14	3.0		5	
	adrenal	32	3.7	34	2.8		41	
9	lung	42	4.0	34	3.4	SD	13	PR
	LN	16	4.8	10	3.0		8	
	LN	15	3.4	15	3.2		15	

*PR* Partial response, *SD* Stable disease, *PD* Progressive disease

^a^PD: appearance of new lesion

**Fig. 1:**
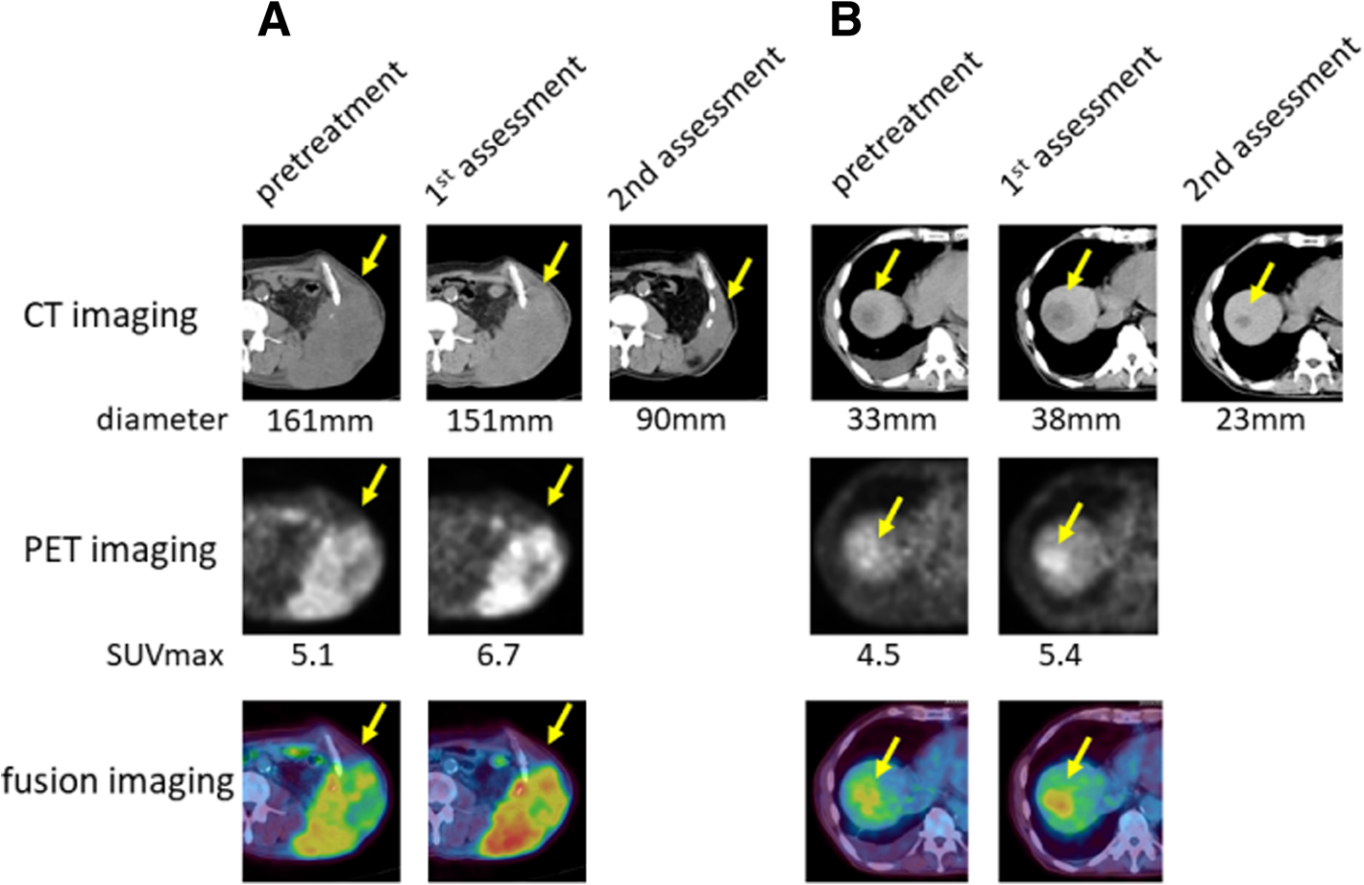
Changes of a subcutaneous and a hepatic lesion in patient #5. **a** Subcutaneous metastasis **b** Hepatic metastasis. CT images (upper lane): The number means maximum diameter of the targeted lesion PET images (middle lane): The number is SUVmax. Fusion images are in the bottom lane

**Fig. 2:**
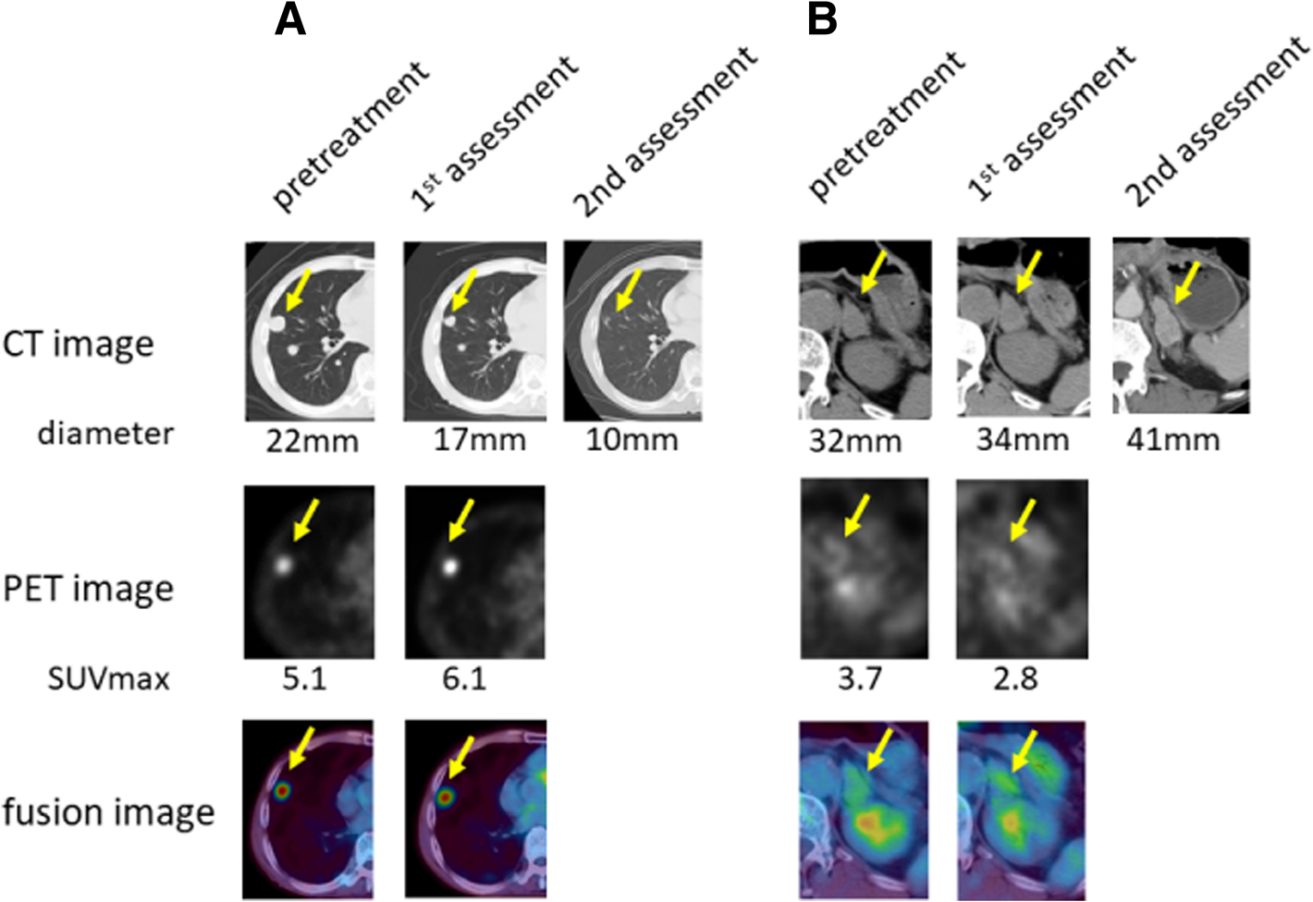
Changes of a pulmonary and an adrenal lesion in patient #8. **a** Pulmonary metastasis **b** adrenal metastasis. CT images (upper lane): The number is the longest diameter of the targeted lesion. PET images (middle lane): The number means SUVmax. Fusion images are in the bottom lane

Interesting findings, different from the reaction to molecular targeted agents, could be seen through the treatment with nivolumab. In patient #5, a large subcutaneous left lower back lesion shrank markedly after an elevation of SUVmax at first assessment (Fig. 1a). A hepatic lesion also responded to treatment after its enlarged diameter and elevated SUVmax was observed at first assessment (Fig. 1b). Additionally, in patient #8, two lung lesions decreased in size while increasing in SUVmax, similar to the subcutaneous lesion seen in patient #5 (Fig. 2a). It is also interesting that another lesion in his adrenal grand had enlarged along with a decline in SUVmax at first assessment (Fig. 2b).

In order to predict the response to nivolumab of each lesions, we divided all lesions to two groups according to the results of the second assessment. Lesions whose diameter decreased ≥30% were defined as responding, and lesions whose diameter did not decrease ≥30% were defined as non-responding lesions. There were 18 responding lesions, and 12 non-responding lesions.

**Fig. 3:**
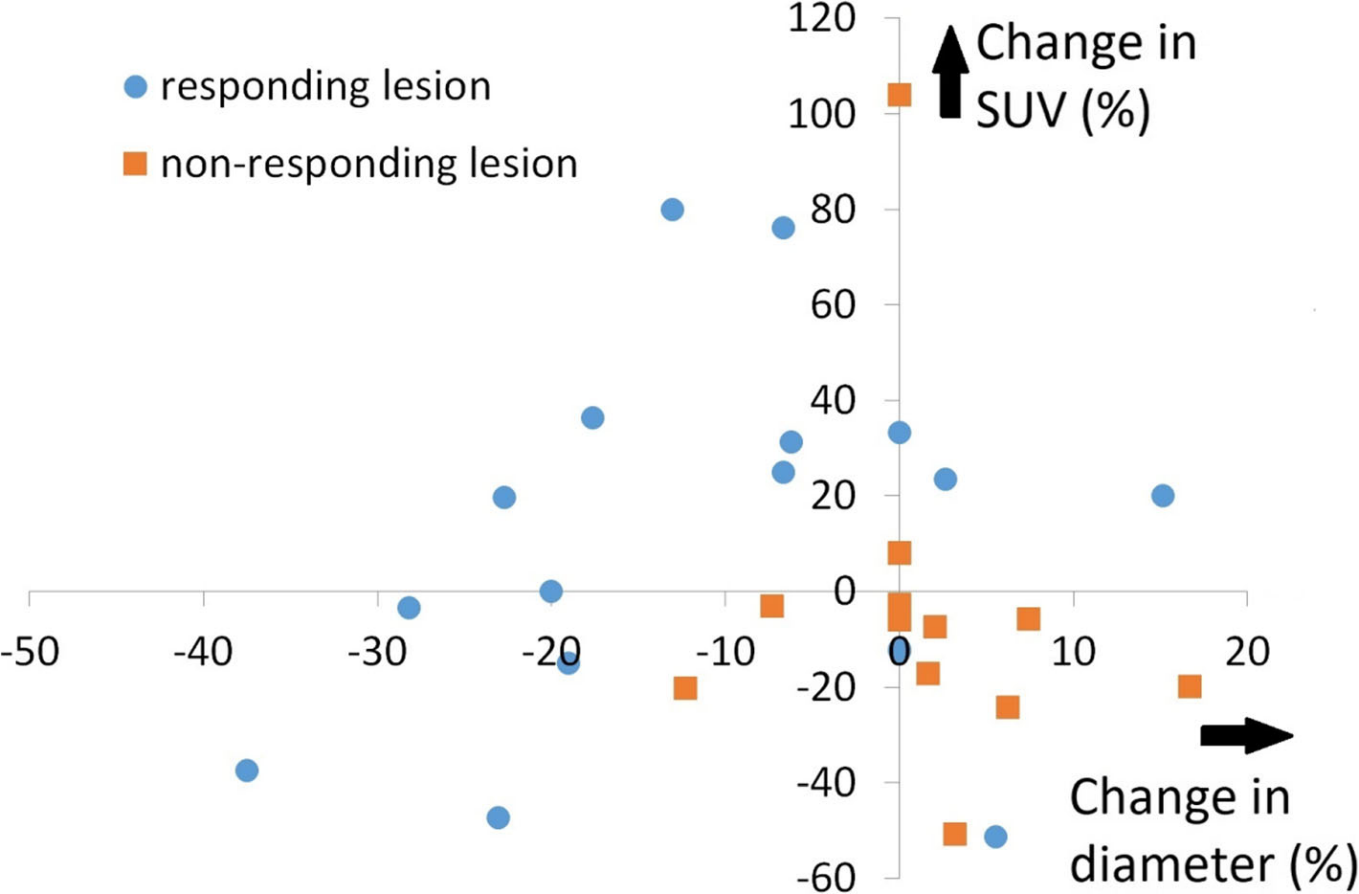
The association among response at second assessment and change ratios of SUVmax and the tumor diameter at first assessment in each lesion. Horizontal axis and vertical axis indicate change in diameter and change in SUVmax at first assessment, respectively. Blue and orange dots indicate responding lesions and non-responding lesion, respectively

Figure 3 is a graph showing the size and SUVmax changes compared. Horizontal axis and vertical axis indicate change in diameter and change in SUVmax at first assessment, respectively. On this graph, the all lesions with decreased diameter and elevated SUVmax showed responding at the second assessment, while majority of lesions with increased diameter and declined SUVmax did non-responding at the second assessment.

Because this study was preliminary and the number of patients enrolled was small, we evaluated the impact of clinical factor of individual targeted lesion, including baseline diameter, baseline SUVmax, metastasis site, elevation of SUVmax at 1month, and decrease in diameter at 1 month on the response at 4 months (responding or non-responding). Table 3 presents the multivariate logistic regression analyses. It revealed that only the elevation of SUVmax at 1 month was an independent predictor (*P* = 0.025, OR: 13.087, 95%CI: 1.373–124.716).

**Table 3:** Multivariate logistic regression analysis of predictive factors for treatment response in each lesion

Parameter	Category	*P*-value	OR	95% CI
Age	―	0.372	0.846	0.586–1.222
Gender	male		1	
	female	0.274	0.116	0.002–5.503
Baseline diameter	―	0.999	1.000	0.960–1.042
Baseline SUVmax	―	0.866	1.069	0.494–2.311
Metastatic site	lung		1	
	non-lung	0.271	3.215	0.403–25.665
Elevation of SUVmax	no		1	
	yes	0.025	13.087	1.373–124.716
Decrease in diameter	no		1	
	yes	0.313	2.889	0.367–22.731

*CI* Confidence interval, *OR* Odds ratio

## Discussion

In the present study, the response to nivolumab in nine patients was assessed with FDG-PET performed both before and 1 month after treatment. Multivariate analysis demonstrated that elevated SUV max at first assessment is a favorable predictor for response to nivolumab.

There are few reports which describe the relationship between immunotherapy and FDG-PET. Kong et al. [24] reported three of eight patients with positive FDG-PET scans treated for melanoma with anti-PD-1 antibodies that were pathologically confirmed to have immune cell infiltrates, with no sign of melanoma cells. It is well-known that inflammation causes false-positive PET findings because inflammatory cells also take up FDG like tumor cells. In addition, immune checkpoint inhibitors enhance glycolysis in T cells [25]. In those cases, it is assumed that elevated SUVmax indicates activated anti-immune response induced by nivolumab. Therefore it can be hypothesized that showing elevated SUVmax is a favorable finding, which is different from our previous reports about molecular targeted therapies [9–11]. Indeed, there are three striking cases shown in Figs. 1 and 2, namely a subcutaneous and hepatic lesion in patient #5 and lung lesions in patient #8. Those lesions became smaller subsequently to SUVmax temporary elevation. It is also interesting that another adrenal lesion in patient #8 whose SUVmax was not increased showed progression at the second evaluation (Fig. 2). It is intriguing that the majority of lesions with increasing SUVmax were judged at second assessment as responding. A pulmonary lesion in patient #5 and lesion in an adrenal grand of patient #6 were classified as a non-responder although SUVmax at first assessment was elevated. However the former did not enlarge and had been stable for at least 77 days between the first and second assessments and the latter showed shrinkage by 25% compared to baseline that is not defined as responding lesion. We have to pay attention to the fact that durable stable disease is a common result with nivolumab [16]. Therefore it is too early to conclude these two lesions as a “true” non-responder at the present moment. It is also impressive that non-responding lesions gathered on the area with declined SUVmax in Fig. 3. These findings support the hypothesis that the stronger inflammatory reaction induced by nivolumab may be a favorable factor of local prognosis. However, an opposite result against our hypothesis was reported recently by a study which investigated the findings of FDG-PET/CT and early response after nivolumab in non-small cell lung cancer (NSCLC) [26]. Although explaining this discrepancy is difficult at this moment, we speculate this difference between NSCLC and RCC originates from their tumor microenvironment (TME). Indeed, RCC is considered to have a unique TME because Th1/CD8 immune cell infiltrates and a high density of mature dendritic cells correlate with favorable prognosis in the majority of solid tumors except for RCCs [27].

In addition, we guess this early time elevation of SUVmax has some possible link to pseudoprogression because both phenomena can be thought to be driven by an activated anti-immune system. We presented a typical course of pseudoprogression in Fig. 1. A hepatic lesion became larger than pretreatment and thereafter started shrinking. As noted in the Introduction, infiltration of immune cells with or without inflammatory edema is a potent mechanism of pseudoprogression. This theory led us to speculate that activation of the anti-tumor immune system precedes changes in tumor volume. We believe that it is more beneficial to know how strong the intratumoral immune system works than how the tumor volume changes. It has been reported that pseudoprogression was observed in 9.7% of melanomas treated with ipilimumab [28], and 5% of NSCLCs treated with nivolumab [29]. In RCC, there is a report that in the population of the Checkmate 025 trial, 13% of patients who received further nivolumab treatment beyond progression had tumor reduction > 30% [30]. Responding to treatment beyond progression is thought to be a favorable prognostic factor in the study although selection bias might play some role. Under the present circumstances in which CT scan is the main modality to evaluate treatment response, more careful assessment is required for clinicians when tumors become larger than pre-treatment. Actually some response criteria validated for immunotherapy require an additional imaging assessment at another time point [28, 31] if patients’ conditions permit. However, from another perspective, the majority of patients who have enlarging lesions despite immunotherapy receive the inefficacious therapy with such a strategy to avoid misclassifying. This is not only a problem of patient survival but also a huge problem of medical economics. Therefore, a novel biomarker to predict the prognosis of a patient treated with immunotherapy should be sought. This study implies that FDG-PET/CT has some usefulness to differentiate pseudoprogression from “true” progression. All non-responding lesions with increasing diameter at first assessment showed declined SUVmax. Finding of declined SUVmax may be a help to discriminate “true” progression from pseudoprogression although there is one responding lesion which increased its diameter and declined SUVmax at 1st assessment. On the other hand, there were some responding lesions with declined SUVmax, for example, a subcutaneous lesion in patient #3 and a pulmonary lesion in patient #6. Although this pattern can be easily acceptable for us because those findings are in line with our previous reports in molecular targeting therapy [9–11], they did not reflect the entire spectrum in this study. Inflammatory response might have appeared before the first assessment in these cases. However, days until first assessment in patient #3 and #6 were 33 and 51, respectively. Therefore, other unknown factors than timing of imaging may exist.

The present work has several limitations. This study includes only nine patients with a total of 30 lesions. This sample size is not enough to conclude a definite relationship between FDG-PET/CT findings and nivolumab. Of course, SUVmax usually elevates when tumor proliferation is accelerated. We have not found an appropriate cut-off value of SUVmax to distinguish patients with aggressive progression from those with inflammatory elevation. Furthermore careful attention should be paid while interpreting the value of SUVmax because it is not standardized among patients or among the organs to which cancer metastasized. Therefore interpersonal or intrapersonal variation of PET-CT must be considered as our group previously described wide variations of SUV in the individual patient with RCC among patients [32], among organs in individual patients [33].

Despite these limitations, we still believe this study is worthy to investigate subjects prospectively with a larger sample size and longer period of observation because early tumor shrinkage is known as a prognostic marker of survival in RCC treatment with molecular targeted agents [34]. Although there are no published data as to nivolumab, this implies that mid-to-long-term prognosis is predictable earlier if predicting short-term outcome 4 months after nivolumab is possible. We believe early time assessment by FDG-PET/CT has the potential to predict prognosis earlier as our present study showed.

## Conclusion

The change of SUVmax of each lesion measured by FDG-PET/CT at an early point with nivolumab treatment can correlate with the short-term local prognosis.

## Abbreviations

anti-CTLA4: Anti-cytotoxic T-lymphocyte-associated protein 4
anti-PD1: Anti-programmed death 1
anti-PDL1: Anti-programmed death ligand 1
FDG-PET/CT: ^18^F-2-fluoro-2-deoxyglucose positron emission tomography/computed tomography
mTOR: Mammalian target of rapamycin
NSCLC: Non-small cell lung cancer
RCC: Renal cell carcinoma
ROI: Region of interest
SUVmax: Maximum standardized uptake value
TKI: Tyrosine kinase inhibitors
TME: Tumor microenvironment
